# Mapping biological process relationships and disease perturbations within a pathway network

**DOI:** 10.1038/s41540-018-0055-2

**Published:** 2018-06-11

**Authors:** Ruth Stoney, David L Robertson, Goran Nenadic, Jean-Marc Schwartz

**Affiliations:** 1 School of Computer Science, University of Manchester, M13 9PT, Manchester, UK; 2 MRC-University of Glasgow Centre for Virus Research, Garscube Campus, Glasgow, G61 1QH UK; 3 Faculty of Biology, Medicine and Health, University of Manchester, Manchester, M13 9PT UK

## Abstract

Molecular interaction networks are routinely used to map the organization of cellular function. Edges represent interactions between genes, proteins, or metabolites. However, in living cells, molecular interactions are dynamic, necessitating context-dependent models. Contextual information can be integrated into molecular interaction networks through the inclusion of additional molecular data, but there are concerns about completeness and relevance of this data. We developed an approach for representing the organization of human cellular processes using pathways as the nodes in a network. Pathways represent spatial and temporal sets of context-dependent interactions, generating a high-level network when linked together, which incorporates contextual information without the need for molecular interaction data. Analysis of the pathway network revealed linked communities representing functional relationships, comparable to those found in molecular networks, including metabolism, signaling, immunity, and the cell cycle. We mapped a range of diseases onto this network and find that pathways associated with diseases tend to be functionally connected, highlighting the perturbed functions that result in disease phenotypes. We demonstrated that disease pathways cluster within the network. We then examined the distribution of cancer pathways and showed that cancer pathways tend to localize within the signaling, DNA processes and immune modules, although some cancer-associated nodes are found in other network regions. Altogether, we generated a high-confidence functional network, which avoids some of the shortcomings faced by conventional molecular models. Our representation provides an intuitive functional interpretation of cellular organization, which relies only on high-quality pathway and Gene Ontology data. The network is available at https://data.mendeley.com/datasets/3pbwkxjxg9/1.

## Pathway networks help capture complex biological function

Molecules within the cell interact to perform biological functions and disruption of these functions often results in disease (e.g., disrupting control of cellular proliferation results in cancer). Molecular networks, such as protein-protein interaction networks, are used to map function; however, these models have limited ability to show certain aspects of biological complexity. For example, proteins may perform different interactions and have distinct functional roles under various cellular contexts or within different tissues. Molecular networks are confined to a single representation of each protein, prohibiting separation of functional roles and muddling the representation of these functions. We assign function to pathways rather than molecules, allowing proteins to be functionally represented within multiple pathways. The model is used to map diseases to their underlying disturbed cellular function.

## Introduction

Cellular processes are carried out by groups of interacting proteins.^1^ Understanding how these spatially and temporally organized sets of interactions lead to biological processes is fundamental to our comprehension of the cell. The conventional approach used to study function has been based on molecular interaction networks, which have improved our understanding of disease,^2–4^ infection,^5^ drug pharmacodynamics,^6^ and evolution.^7^ In this paper, we describe data and networks as “molecular” if they are concerned with interactions between individual biological molecules. This is in contrast to our focus on pathway-level representations, which represent pathway gene sets, with interactions between individual molecules subsumed into the “pathway nodes”. Pathways are considered to collectively participate in biological processes, the functions of individual genes or gene products are not represented.

There are various approaches for studying biological processes using molecular interaction networks. Protein–protein interaction (PPI) data is frequently used to construct networks, in which proteins are shown interacting with functionally related partners. This results in the emergence of functionally related sub-networks known as “functional modules”.^3^ Modular organization of function has been shown to exist across species, and is used to predict gene function.^8,9^ Similar networks have also been generated using co-expression data,^7^ genetic interaction data,^10^ and by combining data types.^11^ However, a disadvantage is that these networks contain false positive and false negative interactions, which may distort our understanding of functional organization.^12–14^

In PPI networks, the edges link each protein to all of its known interacting partners. However, protein interactions are often dynamic, assembling when needed to perform a function, then disassembling.^15–17^ This property is not captured in static networks, where interactions appear permanent in time. Proteins may participate in different functions, depending on the interactions they make in various cellular contexts^18,19^ and subcellular compartments,^20^ making representation of dynamic interactions critical for the accurate portrayal function.^21,22^ To capture the inherently temporal nature of molecular interactions, dynamic models incorporating additional data have been developed. For example, gene expression data have been mapped onto PPI networks to reflect the dynamic nature of protein interactions. Active sub-networks, defined as connected regions of the network that show altered gene expression under particular conditions, can then be identified.^23–25^ Additionally, longitudinally sampled data can be represented using multiple time series networks.^15^ An advantage of this approach is that refining the edges to those present at each time point produces modules that are smaller and more functionally specific.^17^ However, the use of gene expression data fails to capture interactions between proteins that do not have correlated expression.^12^ The correlation between gene expression and protein abundance is also weak.^26–30^

We suggest that the utilization of more reliable data could allow functional models to reach their full potential. In this work, we address the previous limitations by introducing a representation of cellular functions that uses pathways, rather than genes, as the constitutive elements. Pathways are comprised of sets of proteins (and complexes) that interact with each other serially, for example, to form signaling or metabolic pathways. This allows us to group sets of proteins known to interact under particular conditions. Although pathway data is based on molecular interactions within a specific cellular functional context, individual pathways do not include all the interactions that each protein participates in. Pathway data is considered to be more reliable than molecular data since it is based on a consensus reached by biochemists over an extended period of time and repeated experimentation. In this study we have excluded the individual molecular interactions and represented pathways as sets of proteins. As a consequence of this reduction in network complexity, the issues of individual false positive and negative PPIs are avoided, since individual molecular interactions are not represented in the network. The method also avoids gene expression data and the assumption that gene expression represents protein levels. In addition, the pathway model allows proteins to be represented independently in multiple pathways, separating pleiotropic functions. Finally, by simplifying the complexity of PPI networks to a smaller number of pathways, computational analysis becomes less demanding and more accessible.

We present a human pathway network representing global biological function. By incorporating pathways from multiple data sources we aim to maximize functional coverage while minimizing the overlap between pathways. To assess the ability of our network to interpret disease functions, we mapped a broad range of disorders onto the network, before focusing more specifically on cancer. Disease pathway “modules” or clusters are known to form within molecular networks, showing overlap with functional modules.^2,3,31^ Cancer genes have been found to be especially highly connected within PPI networks,^2^ with different types of cancer forming highly connected overlapping modules.^32^ Our representation provides a higher-level view of the pathways and functions affected by disease, without the inaccuracies inherent in molecular-level interaction data.

## Results

### Global functional organization can be represented by a non-redundant set of 1014 pathways

**Fig. 1:**
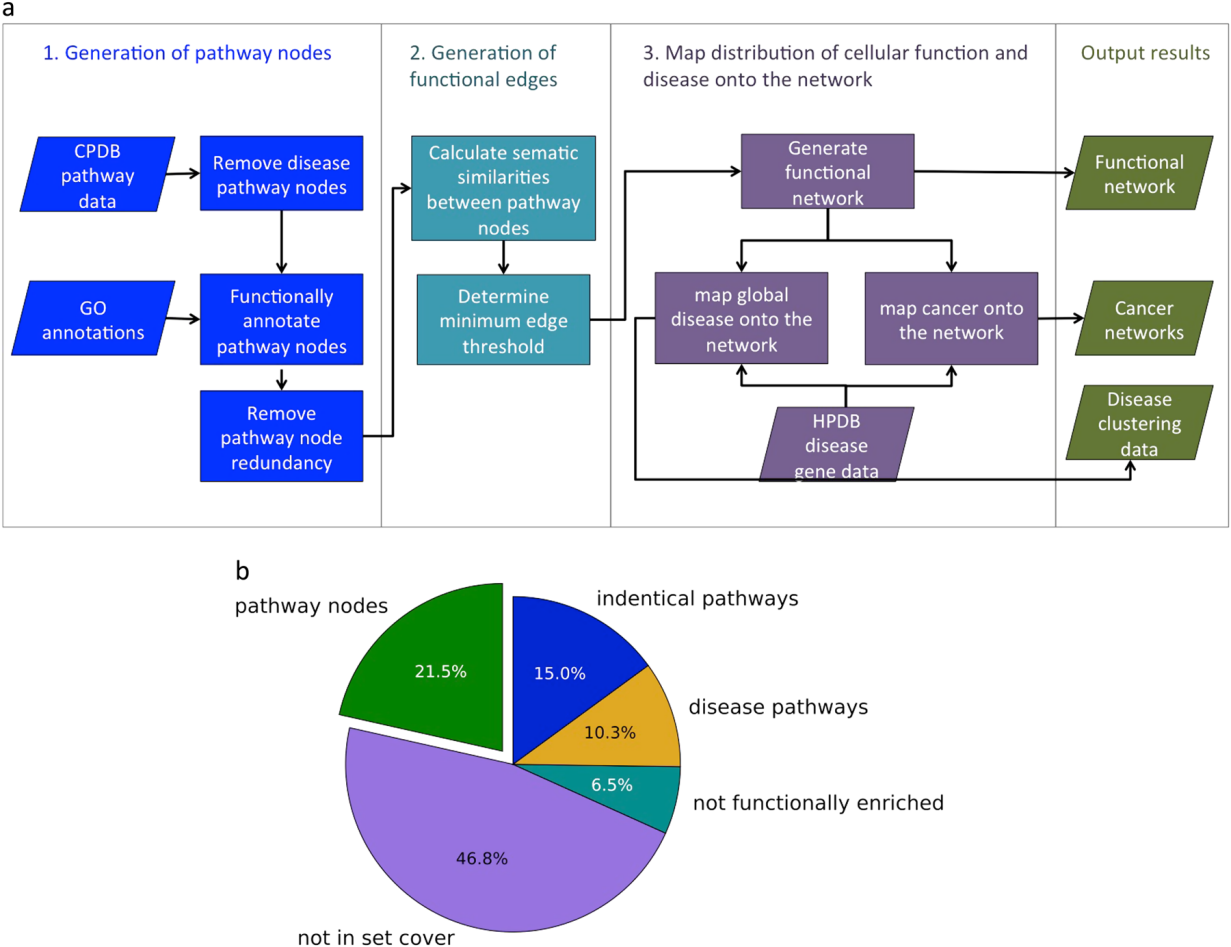
Diagrammatic representation of **a** the project work flow and **b** the proportion of pathways that were removed from the initial data set because they had identical gene sets, were disease pathways, could not be functionally annotated, or were redundant (not in the set cover)

In order to generate a representation of biological processes based on pathways, we first selected a set of non-redundant, functionally annotated human pathways (Fig. 1a). The original dataset contained 4,011 pathways and 11,196 genes. Figure 1b shows the proportion of pathways that were removed at each stage of pathway preparation.

Diseases reflect perturbations of normal cellular functions. In order to represent diseases we first generated a network of pathways showing the cell in a normal, healthy state, then mapped disease genes onto the network. However, the data set contained pathways that were already representations of disease perturbations, and were therefore unsuitable for inclusion in the network. Specifically, disease pathways such as colorectal cancer, asthma and HIV infection were removed from the data set. Drug metabolism and addiction pathways also show the cell in an altered state, therefore drug metabolic pathways such as doxorubicin and statin pathways, and addiction pathways such as cocaine addiction were removed. A total of 484 pathways, with 225 pathways containing disease terms, 30 containing drug terms and 221 addiction terms were removed (see Methods). This only reduced the number of genes in the data set to 10,833.

The Gene Ontology^33^ (GO) assigned a mean of 8.2 terms to each gene (median 5, standard deviation 9.2). Addition of parent terms increased the mean number of GO terms per gene to 75.3 (median 52, standard deviation 71.3). It was necessary to remove 1263 genes, as they did not have experimentally validated GO annotations, resulting in a loss of two pathways. Of the unannotated genes, 4.0% had no Biological Process annotations and 7.6% only had Biological Process annotations inferred from electronic annotation (IEA), which are considered less reliable. We removed 298 pathways with fewer than four annotated genes, as they were too small for enrichment analysis. Enrichment analysis returned at least one high confidence enriched Biological Process GO term (*p*-value < 0.01) for 2514 out of the 2521 remaining pathways. Pathways without enriched GO terms were removed, as functional annotations were required to create edges in the network.

Between 1 and 3459 enriched GO terms were assigned to each pathway (mean 411.8, standard deviation 441.0), using the *p*-value threshold of 0.01. These enriched GO terms varied greatly in their significance and included many similar terms and parent terms. We aimed to generate a network that linked pathways based on the similarity of their enriched GO terms; however, GO terms assigned with low significance had the potential to make spurious connections or link pathways based on highly general terms. To address these issues we selected the most specific set of GO terms available to represent the genes in the pathway. We used the set cover for enrichment analysis algorithm (see “Minimisation of pathway functional profiles” Section) to select the most significant GO terms capable of covering the genes in each pathway [Stoney 2017 submitted], reducing the mean number of GO terms from 411.8 to 4.7 (standard deviation 4.2). These reduced functional profiles provide a precise representation of the pathways’ function without large numbers of similar GO terms or parent terms.

Next we selected a subset of pathways with reduced redundancy and minimal pathway size variability. Pathway size was controlled since the dataset included pathways with up to 2154 genes, which are unhelpful since they lack functional specificity. We used the proportional set cover algorithm (see “Reduction of redundancy between pathways” Section) to reduce redundancy while preferentially selecting pathways with sizes close to the median size of 23.^34^ We allowed the set cover algorithm to finish after 99.95% of the genes had been covered, reducing the number of pathways required to 1014. The only difference between this set cover and the set cover produced to cover 100% of genes was the absence of pathways “gene expression” and “metabolism”. This reduced the maximum pathway size from 1442 (metabolism) to 426 (“generic transcription pathways”), while resulting in the loss of only 4 genes.

**Fig. 2:**
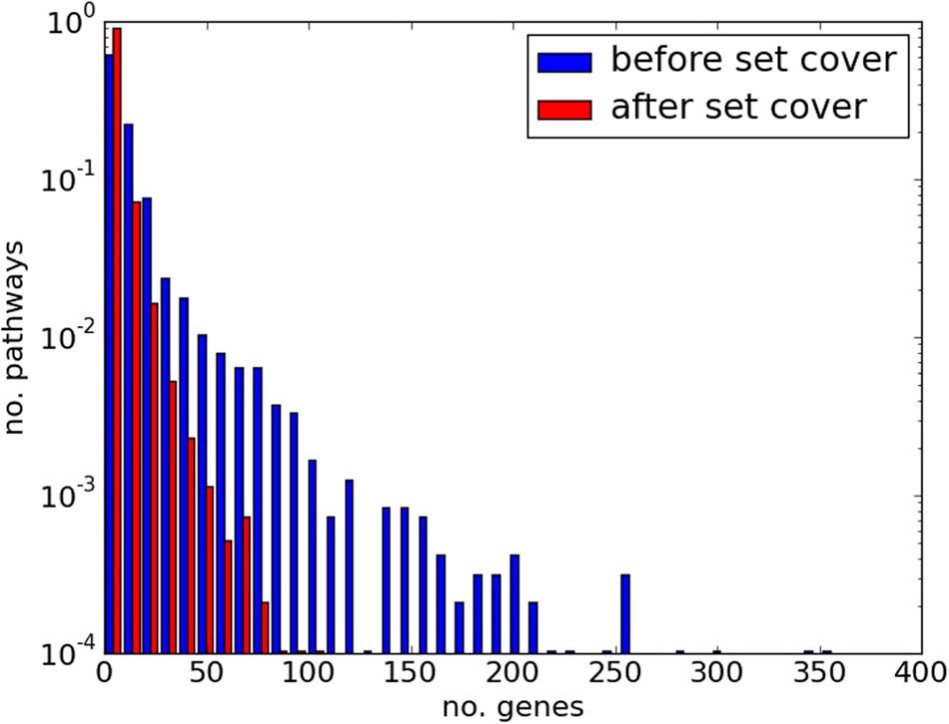
Genes in multiple pathways before and after applying the set cover algorithm. Histogram showing the proportion of the genes in the data set that appear in multiple pathways (indicating redundancy), before and after set cover

Figure 2 shows the ability of the set cover algorithm to reduce redundancy, by displaying the presence of genes in multiple pathways. Prior to redundancy reduction, genes appeared in a mean of 46.0 pathways, with many genes appearing in large numbers of pathways. After set cover, genes appeared in a mean of 4.2 pathways. Genuine cases of pleiotropy are preserved in the remaining overlap, as pathways with minor overlap are not removed. The use of this modified set cover algorithm enables us to use the combined data sources collated by ConsensusPathwayDB (CPDB)^35^ without being undermined by excessive pathway overlap.

### The Wang best-match average is the most suitable metric to measure functional similarity of pathways

**Fig. 3:**
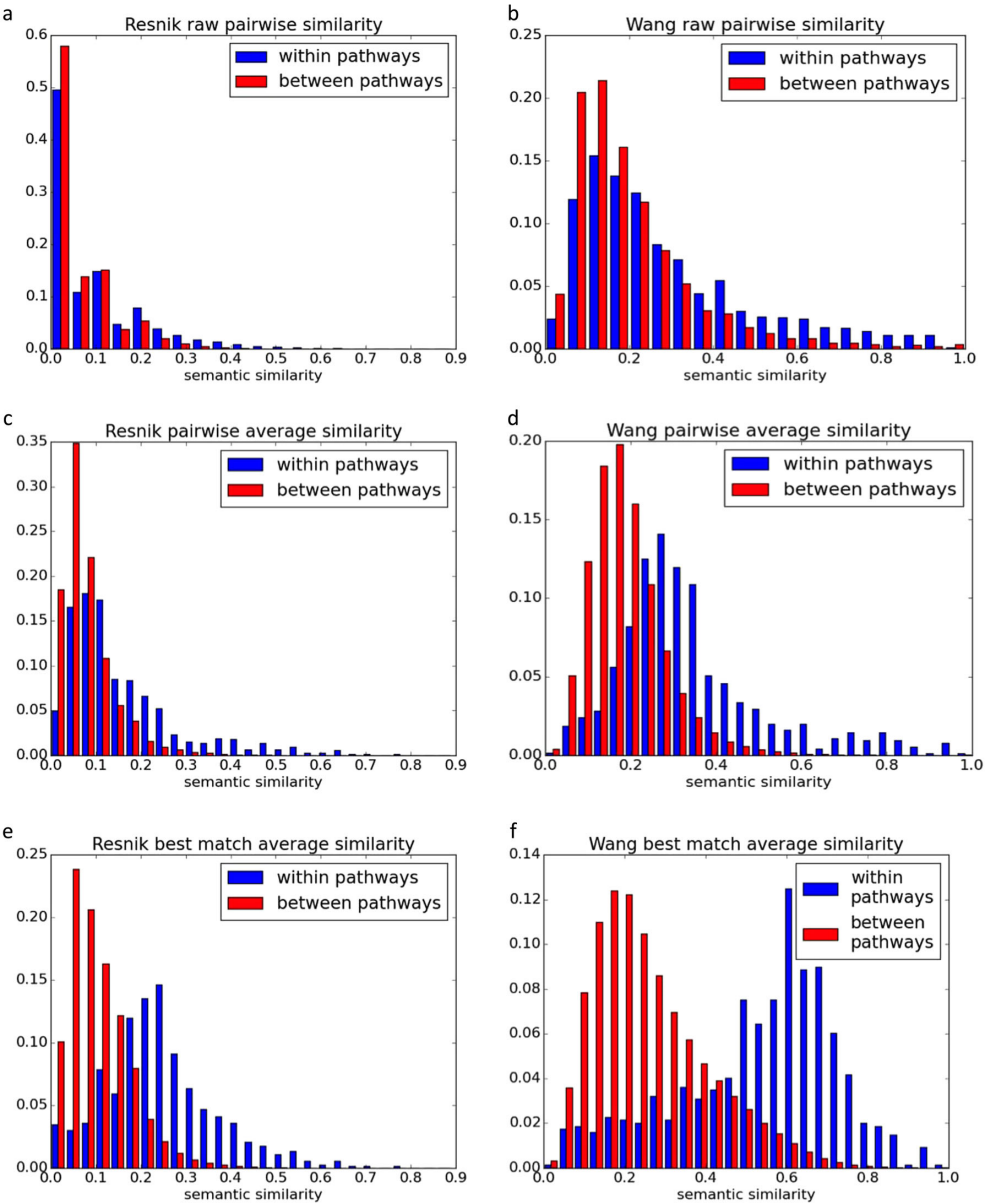
Pathway redundancy across set cover algorithms. Semantic similarities between GO terms in the same pathway (red) and between pathways (blue). The y-axes show the proportion of GO term pairs allocated different semantic distances. **a**, **b** are individual semantic similarity measures taken using the Resnik^36^ and Wang^37^ measures. **c**, **d** are pairwise average distances using the Resnik and Wang measures. **e**, **f** are best-match average distances using the Resnik and Wang measurements

Pathways were linked to form a network based on the similarity of their shared GO terms. We compared the Wang and Resnik methods for measuring distances between GO term pairs (see “Measuring semantic distances between individual GO terms“ Section). The Resnik method measures the distance between two GO terms based on the lowest node shared by both terms within the Gene Ontology topology (referred to as the lowest common ancestor).^36^ The number of genes annotated with the lowest common ancestor term is used to calculate the probability that the GO terms were linked to the lowest common ancestor by chance. In contrast, the Wang method considers all the parent terms of both GO terms, and semantic similarity is calculated based on the proportion of parent terms that are shared by both terms.^37^ The influence of each parent term on the GO terms of interest is considered with greater weights attributed to close parent terms, and with “is-a” links being weighted more heavily than “part-of” links. We then compared the pairwise and best-match average methods for measuring distances between sets of GO terms (see “Measuring the semantic distance between GO sets” Section). To assess the suitability of each method, we identified the approach that gave the greatest difference between the semantic similarities of GO term pairs within pathways, compared to semantic similarities between different pathways (additional information regarding these approaches is given in the “Methods” section). Semantically similar GO pairs are more frequent within pathways than between them, although the difference is small especially when using the Resnik method (Fig. 3a, b).

To generate the pairwise average measure, we calculated the mean similarity between GO terms within each pathway and between each pair of pathways. This increases the distinction between semantic similarities observed between pathway nodes and within pathways. The difference is clearer when distances between GO terms are generated using the Wang measure (Fig. 3d), rather than the Resnik measure (Fig. 3c).

Figure 3e, f show the best-match average similarities between and within pathways. This enhances the distinction between semantic similarities within and between pathways, particularly when the Wang method is used to measure distances between GO terms.

The best-match average typically out-performs the pairwise method when unrelated annotations are allocated to the same pathway or gene.^38^ This is because rather than comparing each GO term to all available terms within each pathway or pathway pair, the best-match average is generated using the most similar GO term pairs. For example if “GO:1” and “GO:2” have a semantic similarity of 0, and are both allocated to “pathway x” and “pathway y”, the pairwise average method will assign an average similarity of 0.5, despite the pathways having identical terms. The best-match average would assign a more intuitive score of 1. The finding that the Wang method outperforms Resnik indicates that pathways are not being assigned a single semantic function but instead are enriched with multiple semantically different GO terms. Clusters of pathways are formed within the network when pathways share at least one function.

The Wang method demonstrably out-performs the Resnik measure, in each recorded instance. To interpret these results, we note that the Resnik measure is based on the lowest common ancestor in the GO ontology capable of covering both GO terms. The score is calculated to describe the specificity of the lowest common ancestor, based on the number of genes associated with the term. A disadvantage of this approach is that it does not consider how far removed each GO term is from the common ancestor.^37^ Therefore two identical generic terms would receive the same score as two highly specific child terms of the generic ancestor, despite their increased difference. The Wang measure considers all ancestral terms shared by two GO terms and reduces the score if the shared ancestors are distantly removed from the terms being compared.^37^ In this way it is better able to distinguish between pairs of general GO terms and pairs of distantly removed GO terms. For these reasons we generated the network using the Wang method in conjunction with the best match average method.

### Pathways linked by shared functionality form a cohesive network

We linked the pathways into a network based on shared functionality, represented by semantic similarity between GO terms. We used the Wang method to calculate functional semantic similarities between each pair of pathways, in order to generate a set of weighted network edges. Inclusion of all the edges generates a highly dense network reflecting the cross-talk between all biological processes, which impedes analysis and structural visualization of the network.

To reduce the number of edges while preserving the underlying structure of the network, we removed weaker edges. To avoid disconnecting large numbers of pathway nodes from the network, we calculated the minimum edge weight threshold for reducing edges while retaining nodes. Using the best-match average technique the optimum threshold to provide the highest number of nodes with the lowest number of edges was 0.56, which conserved 987 nodes (97.1%) and 20,642 edges (4.0%). We used the minimum edge threshold to select a set of edges to construct the network. The resulting network was highly modular with a clustering coefficient of 0.593. Random networks generated to preserve the degree distribution had clustering coefficients ranging from 0.186 to 0.205 (mean 2.01), indicating that the pathway network is more modular than expected from chance. For the 987 nodes in the network, 974 were located within the largest connected component. Application of the Kolmogorov-Smirnov test revealed that, in contrast to many molecular networks,^39–41^ the degree distribution of the pathway network did not follow a power law distribution (*p* < 0.05).

**Fig. 4:**
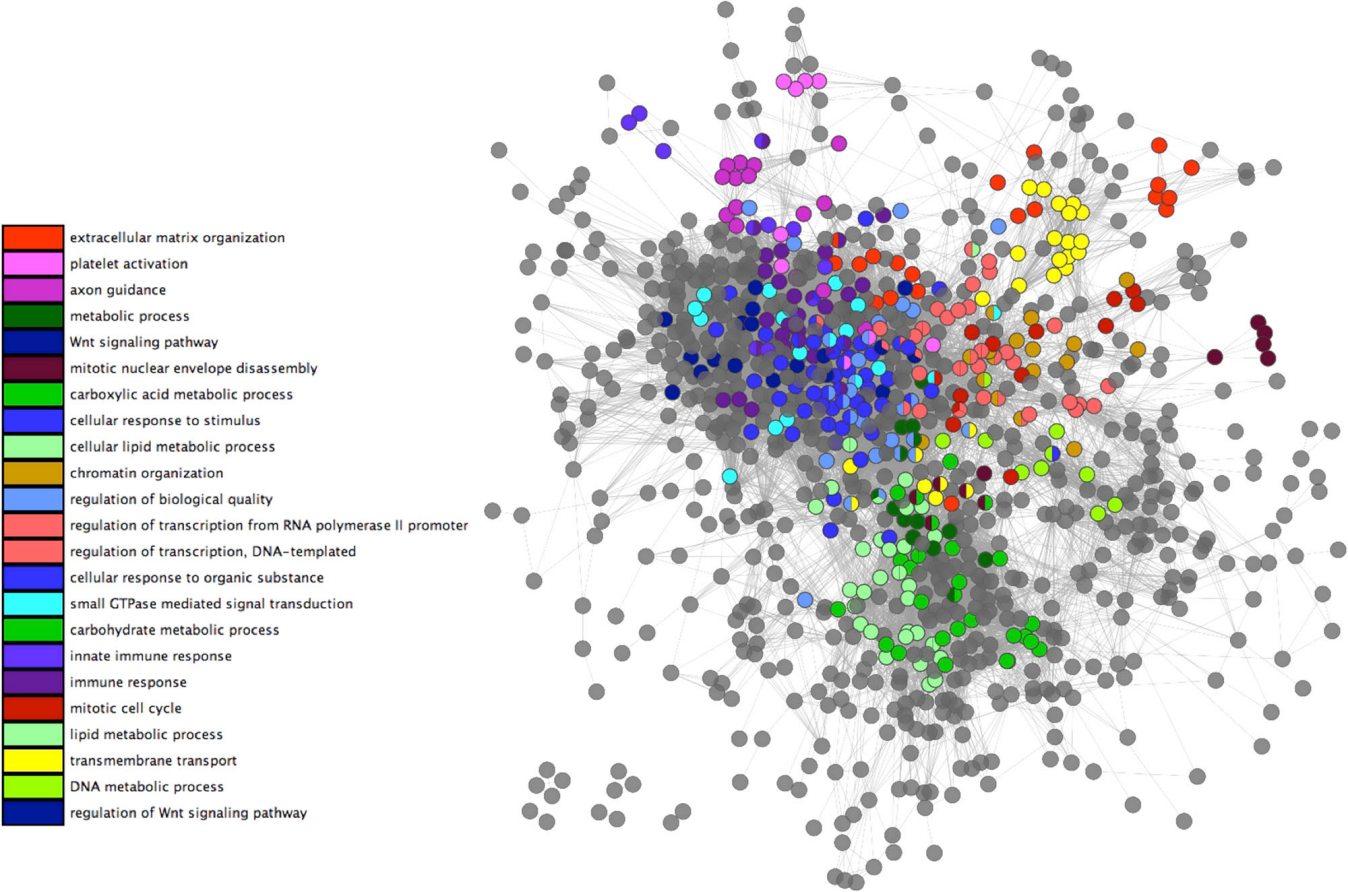
Major functional clusters in the human pathway network. Weighted network of pathways, linked by shared functionality. Edges were generated using the Wang^37^ best match average method to link pathways biased on their functional profiles, using a minimum weight cut off of 0.56

Figure 4 shows the network with a sample of GO terms highlighted to illustrate some of the functions represented. Within the network two major functional pathway modules relating to metabolism and signaling can be observed. A DNA metabolic process module links transcription processes, chromatin organization and mitotic cell cycle to metabolism. Immune responses are tightly clustered besides signaling and cellular responses to stimuli. Axon guidance has nodes in the immunity network region, reflecting it's role in the primary immune response.^42^

### The functional network enables identification of disease pathway modules

We used enrichment analysis to assign 404 OMIM diseases to 219 pathways, using a *p*-value threshold of 0.01. By focusing on diseases (e.g., cystic fibrosis) rather than phenotypes (e.g., chronic lung disease, elevated sweat chloride, and hepatomegaly) we capture the range of symptoms induced by disorders.

**Fig. 5:**
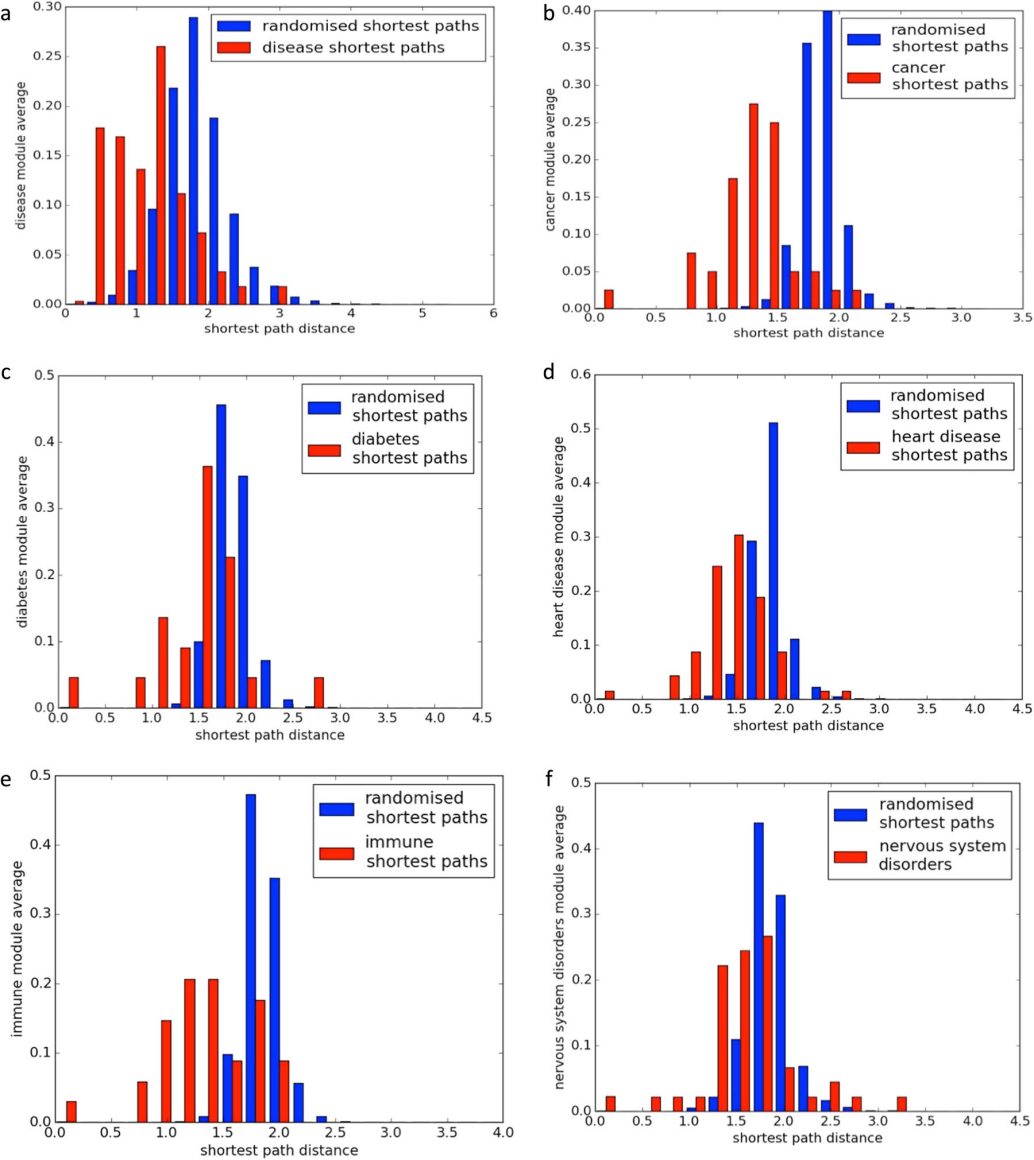
Disease module connectivity. **a** Shortest paths between nodes enriched for the same disease and randomized disease nodes. **b** Shortest paths between nodes enriched for the same type of cancer and randomized cancer nodes. Graphs **c**–**f** show the distribution of shortest paths and randomized shortest paths for diabetes and obesity, heart disease, immune disorders, and disorders of the nervous system

To test the hypothesis that disease nodes form highly linked disease pathway modules we measured the shortest paths between disease nodes. Figure 5a shows the distances between nodes with shared diseases, compared to an equal number of random pathways. Shortest paths between randomized nodes formed a roughly normal distribution, whereas shortest paths between disease nodes tended to be shorter, indicating that disease nodes are close within the network. To confirm the significance of the distributions we performed a one sample Kolmogorov–Smirnov test, which returned a *p*-value of <0.01.

### Disease pathway modules

We identified 166 pathways enriched with cancer genes at a *p*-value of <0.01. These were comprised of 39 types of cancer affecting a range of cell types. Many pathways were enriched for multiple cancer phenotypes (mean 3.3). The pathway associated with the most cancer types (17) was “extracellular vesicle mediated signaling in recipient cells”, which contains cancer causing genes including *WNT, EGFR, RAF, NRAS*, and *KRAS*, and is upstream of pivotal cancer pathways.^43^ Other pathways associated with high numbers of cancers were the “*RAC1 PAC1 P38 MMP2* pathway” containing *MAPK, ERK, KRAS, RAC, RAS* genes and “copper homeostatis” which has been found to be relevant to multiple tumor types and is being trialed as a chemotherapy target.^44^

To assess the claim that cancers cluster within particular network regions, we measured the shortest paths between cancer nodes within the network (Fig. 5b). The Kolmogorov–Smirnov test was applied to confirm the significance of the observed cancer clusters (*p*-value < 0.01). To assess whether the formation of disease clusters was unique to cancer we also tested pathway sets related to diabetes and obesity, disorders of the nervous system, immune system and cardiovascular system (Fig. 5c–f). In each instance, pathways associated with a shared disease phenotype were closer within the network than expected at average (Kolmogorov–Smirnov test *p*-value < 0.01).

**Fig. 6:**
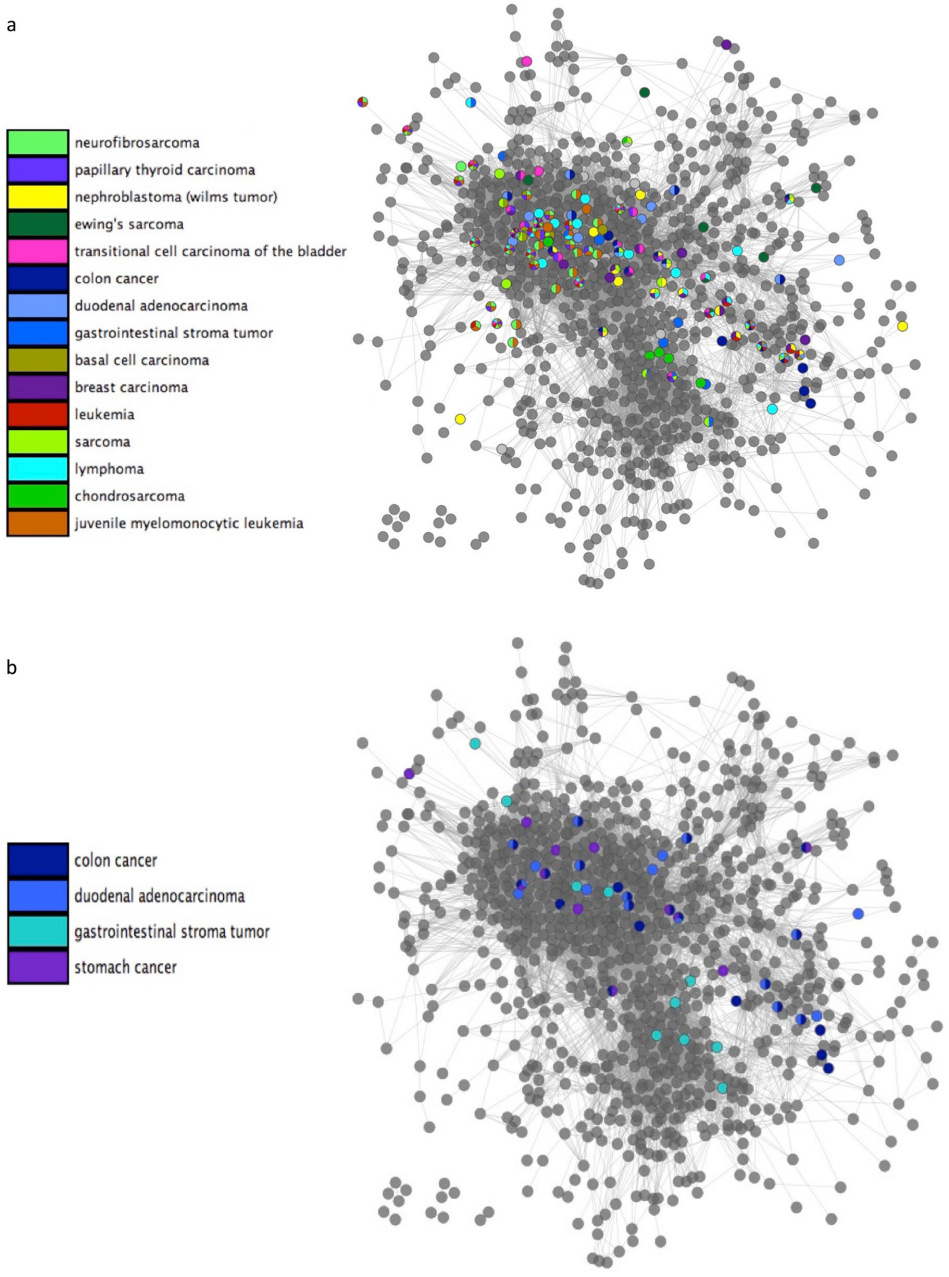
Distribution of cancer pathways. **a** Functional pathway network showing the distribution of pathways associated with common cancer types (in the data set). **b** Distribution of colon cancer, duodenal adenocarcinoma, gastrointestinal stroma tumor, and stomach cancer

We examined the distribution of cancer within the network. Figure 6a shows the topological position of a sample of cancers affecting high numbers of pathways in the dataset. Cancer pathways can be seen clustering primarily within the signaling, immune response and DNA process network regions. The signaling and immune network region is the most densely populated with cancer nodes, including sarcoma pathways, juvenile leukemia, and neurofibrosarcoma. Cancer nodes also cluster in the region concerned with DNA metabolism, response to stimulus, and transcriptional control. Several breast cancer and nephroblastoma pathways are also prevalent in this region.

The distribution of disease pathways within the network can indicate similarities and common risk factors between related distorders. To demonstrate this application, we present the distribution of gastorintestinal cancer pathways onto the network (Fig. 6b). Some pathway overlap between gastrointestinal stroma tumor, colon cancer and duodenal adenocarcinoma is observed, with shared pathways corresponding to common cancer processes and risk factors. The common risk factors of duodenal adenocarcinoma and colon cancer are gastrointestinal polyps and chromic inflammatory bowel disease.^45,46^ Correspondingly, within the network both cancers are found to be enriched in BMP signaling pathways, which have been shown to affect gastric inflammation.^47^ DNA repair, cell cycle, extracellular vascular mediated signaling and RAF activation pathways were frequently shared by multiple cancer types.

## Discussion

The use of molecular networks to study biological processes has been highly insightful. However, limitations with molecular interaction data and issues representing multi-functional genes make the development of alternative methods a necessity. We have constructed a functional network from existing pathway data and biological process annotations. The pathway network portrays a higher-level representation of the organization of biological processes, composed of functional pathway modules. Clustering methods used in molecular networks identify specific relationships in which each node shows a high density of interactions with all of the other nodes in the cluster. These methods are less suitable for identifying linear functional structures, in which chains of nodes interact without having a high clustering coefficient. Other studies have also approached the issue that network structures, other than clusters, may represent functional modules.^48^ Pathways are sets of interactions, which were manually curated to adopt the most appropriate shape for the data, therefore they represent coherent functions independently of the molecular topology.

Mapping diseases onto molecular interaction networks has contributed towards the elucidation of disease mechanisms,^4^ identification of new disease-associated genes^3^ and indication of potential drug targets.^49^ However, gene mutations can be phenotypically diverse, such as *AKT1*, which is associated with schizophrenia, colorectal cancer, ovarian cancer and breast cancer.^50^ Further evidence suggesting that diseases may act independently within different pathways comes from the finding that many disease pairs with shared genes do not show significant co-morbidity.^51^ Phenotypically diverse genes may also interact with different partners in different tissues, for example *AKT1* participates in a range of interactions dependent on tissue type,^50^ further supporting the hypothesis that the results arise from the gene acting in different pathways. This shows that pleiotropy allows genes to be involved in multiple disorders in different contexts, demonstrating that pathways are better suited than molecular networks to map functional perturbations occurring in diseases. It should be noted that although string matching effectively removes a majority of disease pathways from the network, some disease pathways may remain within the network, making identification of the normal functions affected more difficult. In addition, removal of pathways containing disease, drug and addiction terms may have resulted in the loss of some pathways representing normal, healthy biological processes. However, the remaining pathways cover 97% of the initial gene set, reducing the impact of this concern.

Examining the similarities and differences between diseases is necessary to assess the shared applicability of knowledge and drugs. Our map makes these relationships immediately obvious. This method can be generalized to facilitate understanding of any group of disorders or phenotypes.

## Methods

To generate the data for the network, we selected a low-redundancy set of human pathways, representing healthy biological processes. We assigned function to the pathway nodes and generated edges building on a method previously developed in yeast.^22,34^ Finally we looked at the biological processes attributed to each area of the network and investigated the distribution of disease pathway nodes.

### Generation of pathway nodes

Pathways were downloaded from CPDB on 24^th^ Sept 2015,^35^ providing a dataset of 4011 unique pathways containing 11,196 genes. CPDB collects and compiles data from major pathway databases such as KEGG, Reactome and WikiPathways. Of these pathways, 706 were exact duplicates and were removed. To be included in the network, pathways had to meet the following three requirements, they: represent the cell in a normal, healthy state (pathways depicting to disease perturbations, addiction and drug metabolism were removed, see “Removing disease pathway nodes” Section); had high confidence enriched GO annotations (see “Functional annotation of pathway nodes” Section); and belong to a reduced redundancy subset (see “Reduction of redundancy between pathways” Section).

#### Removing disease pathway nodes

To generate the functional network, we identified a set of pathways representing normal functions. We removed diseases by searching for disease terms within the pathway names (listed in Supplementary Data 1), as they do not show the cell in a normal, non-diseased state. This was considered necessary since in the later stages of the study, we mapped diseases onto the pathway network, to reveal functions affected by particular diseases. The inclusion of disease pathway nodes would distort this distribution, as well as contributing to pathway redundancy.

#### Functional annotation of pathway nodes

To generate the network, we required functional profiles for each pathway node. We assigned high confidence GO terms to each gene, before using enrichment analysis to annotate pathways. Any pathway node that could not be functionally annotated was removed, as we could not calculate their similarity to other pathway nodes to establish network edges.

##### Functional annotation of pathway genes

The Gene Ontology provides Biological Process annotations for individual genes, along with information specifying how annotations are generated.^33^ We assigned high confidence Biological Process GO annotations to genes (downloaded 24^th^ Sept 2016), discarding electronically annotated (IEA) terms as they are of lower confidence than experimentally validated terms.^38^

We were able to assign high confidence, curated GO annotations to 88% of the genes in normal cellular pathways. We also added all non-IEA parent terms to the GO terms allocated to each gene, since for every GO term associated with a gene, all of the GO term’s ancestors apply.^52^ To meet the minimum criteria for enrichment analysis, each pathway must contain at least four genes with Biological Process GO annotations.^53^ Any pathways that contained fewer than four annotated genes were removed.

##### Functional enrichment of pathway nodes

Functional enrichment analysis was carried out using the R package clusterProfiler.^54^ Enrichment analysis returned large sets of GO terms with *p*-values below 0.01 for pathway nodes (mean of 412.0 GO terms per pathway), using the Benjamini and Hochberg correction^55^ for multiple testing.

#### Minimization of pathway functional profiles

We generated minimal sets of enriched high confidence GO terms to represent all of the genes in each pathway node, by removing similar enriched GO terms. We have previously described a set cover algorithm that reduced redundancy from enrichment analysis data,^34^ which we use here to remove redundancy from each pathway’s enriched GO terms. The most specific/enriched GO terms that describe the function of all the genes in each pathway are identified and retained. GO terms describing the same genes with a lower level of significance are discarded, resulting in a reduced functional profile (Supplementary Figure 1A). Note that only the non-IEA GO terms associated with each pathway’s genes will be selected for inclusion in the minimal profile.

#### Reduction of redundancy between pathways

Following the removal of disease and functionally unannotated pathway nodes, all remaining pathway nodes were suitable for use in the network. However, because the data source used was highly inclusive, incorporating pathways from all areas of study, high levels of pathway overlap were present. An extensive effort was made to remove as much data duplication as possible, while preferentially selecting moderately sized pathways. Removal of redundancy was necessary since we aimed to generate a network in which linked nodes represent functional cooperation between distinct pathways.

We have previously described methods using set cover theory to reduce redundancy in pathway data sets.^34^ These combinatorial optimization algorithms identify subsets of pathways that cover all the genes in the dataset. As the data set contained pathways with up to 2,154 genes, controlling the pathway size was critical for preserving functional specificity. We therefore selected the proportional set cover algorithm^34^ as it controls pathway size variability while minimizing pathway overlap. This algorithm iteratively selects the sets containing the highest number of uncovered elements. If multiple sets contain equal numbers of uncovered elements, the set whose size is closest to a predefined target number (such as the average pathway size) is selected. This continues until all of the elements in the dataset are covered (Supplementary Figure 1A).

We note that significant improvements in the algorithm’s ability to control pathway size variability have been observed when the algorithm was allowed to cover “most” rather than all of the genes in the dataset.^34^ We found that allowing the set cover method to cover 99.95% rather than 100% of the genes in the dataset reduced the maximum pathway size from 2,154 to 426. Large reductions in pathway redundancy were also observed (see “Results” Section).

### Generation of edges

To generate the edges in the network, we measured the semantic similarity of each pair of pathway nodes based on their associated GO terms in the minimized functional annotation profile (see “Minimisation of pathway functional profiles” Section). These values, between zero and one, formed the basis of the network edges.

#### Semantic similarities between pathways

To calculate the semantic similarity between pairs of pathways, we first needed to measure the similarity between pairs of GO terms. This was necessary since the methods used to generate semantic similarity are not suitable for highly redundant sets of GO terms (see “Measuring the semantic distance between GO sets” Section). Since pathways are enriched with multiple GO terms, we established the most suitable method for comparing GO sets. Various measures are available for measuring the distance between GO terms and GO term sets.^36,37,56^ We selected our method based on its ability to comply with the assumption that GO terms within pathways should be more closely related than GO terms between different pathways.

##### Measuring semantic distances between individual GO terms

Of the various methods available to measure the distance between two GO terms, the Resnik^36^ and Wang^37^ measures have been shown to outperform other methods in previous studies.^38^ We therefore implemented these methods using the R package GOSemSim.^57^ Supplementary Figure 1B provides an overview of these methods.

##### Measuring the semantic distance between GO sets

To calculate the similarity between pathways, we tested two approaches: the pairwise average method and the best-match average.^38^ The pairwise average method measures the similarity between every pair of GO terms between two pathways and then calculates the mean. The best-match average records the similarity between each GO term in the first pathway and the closest GO term in the second pathway. It then performs the symmetric calculation, before generating a mean distance based on both sets of scores. This produced a semantic distance between every pair of pathways generating a complete network. The complete network was impractical for global analysis, therefore edges were reduced (see “Pruning edges between pathway nodes” Section).

#### Pruning edges between pathway nodes

Our network links pathway nodes using weighted edges based on their similarity. We aimed to reduce the number of edges in the network to show only the most significant functional links between the pathways. We generated a range of 50 thresholds between zero and one and calculated the proportion of nodes and edges retained by each. By subtracting the proportion of nodes retained by each threshold by the proportion of edges retained, we identified the threshold that linked the maximum number of nodes into the network using the fewest edges. Following network generation the degree distribution of the network was subjected to power law analysis using the R igraph package version 1.0.0.^58^ The clustering coefficient was calculated using Cytoscape 3.2.1^59^ and for comparison ten randomized networks were generated using Network Randomizer 1.1.3.^60^

### Mapping the distribution of biological function and disease onto the network

#### Mapping global diseases onto the network

We mapped diseases on to our network using the Human Phenotype Ontology (HPO) disease data, downloaded on the 30^th^ of April 2016. This dataset contained 293,556 disease gene annotations for hereditary and non-hereditary disorders; this data includes both OMIM diseases such as “migraine, familial hemiplegic, 1; FHM1” and phenotypes, such as “visual hallucinations”. Using the Fisher’s exact test to map disease terms onto pathway genes we revealed 1061 disease annotations associated with at least 4 genes to ensure significant enrichment analysis across multiple pathways. We used the Fisher’s exact test to identify 219 pathways associated with 404 OMIM diseases, using a *p*-value threshold of 0.01.

#### Finding the shortest paths in disease sub-networks

To test whether diseases tended to cluster within the network, we measured the shortest paths between pathways associated with each disease using NetworkX.^61^ This algorithm calculates the shortest path between two nodes. This measure conventionally uses distance rather than similarity. We compared these results to sets of shortest paths generated from sets of random nodes. We selected randomized sets of nodes of equal size to the set of disease nodes. We repeated this method 100 times for each disease.

#### Mapping disease systems onto the network

We selected cancers by searching for the terms: *cancer,* *tumor, tumour, melanoma, carcinoma, leukemia, lymphoma*, and *sarcoma* in the set of HPO phenotypes enriched to a *p*-value of 0.01. We mapped the locations of 166 cancer related pathways onto the network and examined associations with biological processes. To measure the tendency of cancers to cluster within the network, we measured the shortest paths between pathway nodes with the same phenotype (see “Finding the shortest paths in disease sub-networks” Section).

Similar pathway sets were generated for the immune diseases, cardiovascular diseases, disorders of the nervous system, obesity and diabetes, using string searches for common disorders and anatomical terms. Supplementary data 2 contains the full set of search terms and generated disease pathways for each biological system.

#### Code availability

The code required to generate these results is available at https://data.mendeley.com/datasets/hn6t9hjfry (see declarations for full break down of main results and data files).

### Availability of data and materials

Data files can be found at https://data.mendeley.com/datasets/37pkdchpf9/1. The code and data files results are available at (https://data.mendeley.com/datasets/hn6t9hjfry, https://data.mendeley.com/datasets/mnjw6rcmcc/1). The file “main results” contains all the main results including the set cover pathways, pathway functions, pathway disease annotations, all semantic distances and the Cytoscape file containing the network (https://data.mendeley.com/datasets/3pbwkxjxg9/2).

Files containing the code are available along with the CPDB and Gene Ontology files used. OMIM files were not included for licencing reasons. The main project is written in Python however it requires output from an (included) R script.

## Electronic supplementary material

data 1

Supplimentary figure 1

Supplementary Data 2

## Electronic supplementary material

**Supplementary information** accompanies the paper on the *npj Systems Biology and Applications* website (10.1038/s41540-018-0055-2).
