# Supplementary material for: Mapping biological process relationships and disease perturbations within a pathway network
Source: NPJ Syst Biol Appl. 2018 Jun 11;4:22. doi: 10.1038/s41540-018-0055-2 (PMC5995814; doi:10.1038/s41540-018-0055-2)
Supplement: Supplementary file 1 — data 1 [file 41540_2018_55_MOESM1_ESM.docx]

# Supplementary materials

Supplementary Data 1 Disease strings. Disease search terms used to remove disease pathways.

| action pathway | constitutive | immunodeficiency | salmonella |
| --- | --- | --- | --- |
| addiction | cystic fibrosis | infection | schizophrenia |
| aflatoxin | cytoma | infertility | sclerosis |
| alcohol | deficiency | influenza | shigellosis |
| allograft rejection | depression | intolerance | spinal cord injury |
| amphetamine | diabete | legionellosis | staphylococcus |
| amyloids | diphtheria | leishmaniasis | substance abuse |
| anaesthetic | disease | leukemia | susceptibility |
| anthrax | disorder | longterm depression | syndrome |
| anxiety | disulfiduria | lupus | tetanus |
| argininemia | drug | malaria | tetanus |
| arsenate | epsteinbarr | measles | toxin |
| arthritis | escherichia | melanoma | toxoplasmosis |
| asthma | ethanol | morphine | trypanosomiasis |
| bacterial | fanconi anemia | mutant | tuberculosis |
| biotin | glioma | nicotine | tumor |
| bipolar | hepatitis | obesity | tumour |
| blastoma | hereditary | obsess | uria |
| botulinum | heroin | pathogenic | vibrio |
| cancer | herpes | pathological | viral |
| carcinoma | hiv | pertussis | virion |
| cardiomyopathy | htlvi | pharmacodynamics | virus |
| carnosinemia | hypertrophy | pharmacokinetics | west nile |
| cholerae | hypophosphatasia | protection |  |
| clostridium | iasis | resistance |  |
