# Supplementary figures and images for: Mapping biological process relationships and disease perturbations within a pathway network
Source: NPJ Syst Biol Appl. 2018 Jun 11;4:22. doi: 10.1038/s41540-018-0055-2 (PMC5995814; doi:10.1038/s41540-018-0055-2)

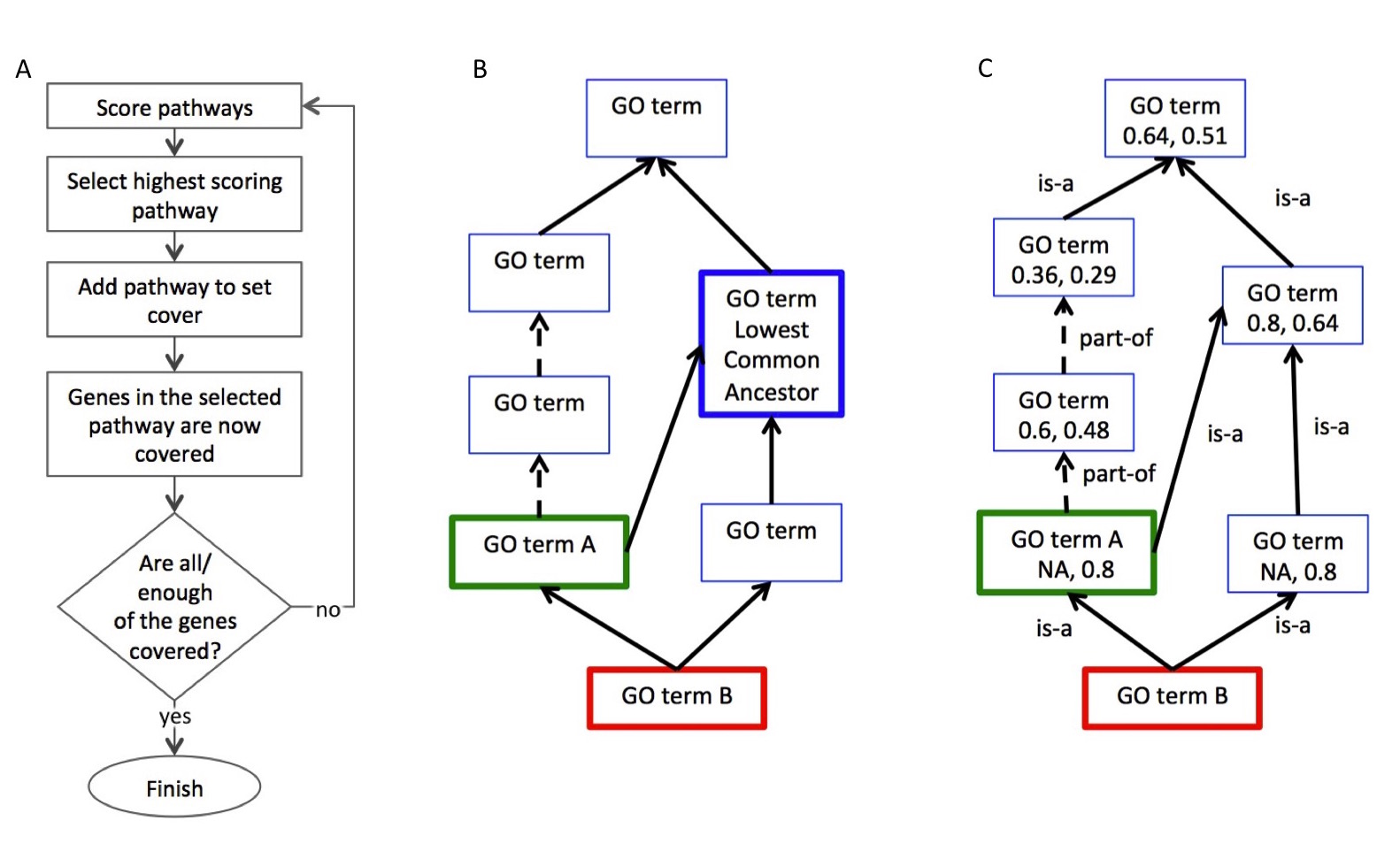

Supplement: Supplementary file 2 — Supplimentary figure 1 [file 41540_2018_55_MOESM2_ESM.jpg]
